# *Schistosoma* Infection and *Schistosoma*-Derived Products Modulate the Immune Responses Associated with Protection against Type 2 Diabetes

**DOI:** 10.3389/fimmu.2017.01990

**Published:** 2018-01-17

**Authors:** Chun-Lian Tang, Zhi-Ming Liu, Yan Ru Gao, Fei Xiong

**Affiliations:** ^1^Department of Science and Education, Wuchang Hospital, Wuhan, China; ^2^Medical Department, City College, Wuhan University of Science and Technology, Wuhan, China; ^3^The Center for Biomedical Research, Tongji Hospital, Tongji Medical College, Huazhong University of Science and Technology, Wuhan, China

**Keywords:** *Schistosoma*, type 2 diabetes, immune regulation, macrophages, type 1 helper T cells

## Abstract

Studies on parasite-induced immunoregulatory mechanisms could contribute to the development of new therapies for inflammatory diseases such as type 2 diabetes (T2D), which is a chronic inflammatory disease characterized by persistent elevated glucose levels due to insulin resistance. The association between previous *Schistosoma* infection and T2D has been confirmed—*Schistosoma* infection and *Schistosoma*-derived products modulate the immune system, including innate and acquired immune responses, contributing to T2D disease control. *Schistosoma* infections and *Schistosoma*-derived molecules affect the immune cell composition in adipose tissue, dampening inflammation and improving glucose tolerance. This protective role includes the polarization of immune cells to alternatively activated macrophages, dendritic cells, eosinophils, and group 2 innate lymphoid cells. Furthermore, *Schistosoma* infection and *Schistosoma* products are effective for the treatment of T2D, as they increase the number of type 2 helper T cells (Th2) and regulatory T cells (Tregs) and decrease type 1 helper T cells (Th1) and type 17 helper T cells (Th17) cells. Thus, our aim was to comprehensively review the mechanism through which *Schistosoma* infection and *Schistosoma* products modulate the immune response against T2D.

## Introduction

Schistosomiasis results from parasitization by worms of the genus *Schistosoma*, of which *S. haematobium, S. japonicum*, and *S. mansoni* are the most widespread species. *Schistosoma* have developed several strategies to manipulate the host immune system to survive, and studies on parasite-induced immunoregulatory mechanisms might contribute to the development of new therapies for inflammatory diseases. Arthritis (1), severe asthma (2), autoimmune encephalomyelitis (3), and inflammatory bowel disease (4) can be ameliorated or prevented by *Schistosoma* infection or *Schistosoma* products. Helminths can modulate the composition of the intestinal microbiota, which has been identified as one potential regulator of obesity and diabetes (5). The transfer of intestinal microbiota from *Heligmosomoides polygyrus*-infected mice was sufficient to protect against allergic asthma, which confirms that the intestinal microbiome is a critical regulator of helminth-mediated protective effects (6). It is likely that helminth infections alter the gut microbiome during diabetes. Actually, animal models have shown that chronic exposure to *S. mansoni* prevents the onset of type 1 helper T cells (Th1)-mediated diseases such as diabetes mellitus (7–9) and *S. mansoni* egg soluble antigen prevents diabetes in NOD mice by inducing type 2 helper T cells (Th2) and Treg responses (10, 11).

Type 2 diabetes (T2D) is a common and serious inflammatory disease characterized by persistent elevated glucose levels due to insulin resistance (IR). This leads to kidney failure, lower-limb amputations, and adult-onset blindness. The healthcare and economic costs associated with T2D are enormous. T2D can be treated, and its consequences can be alleviated or delayed with proper diet, physical activity, medication, and regular screening and treatment of complications. Some studies have suggested that IR can be improved through immunotherapy (12, 13). Data from animal models have shown that helminth infections can reduce IR by modulating the immune system. *Schistosoma* induces metabolic alterations in many metabolic tissues such as white adipose tissue (AT), a highly dynamic organ that responds to nutrient and environmental stress, liver, skeletal muscle, pancreas, and hypothalamus, and also mediates immunoregulation to protect the host against T2D. There is a negative association between the proportion of children per country requiring preventive chemotherapy for soil-transmitted helminths and the prevalence of diabetes (14). The prevalence of T2D is considerably lower in people with previous *Schistosoma* infection (PSI) than in people with no PSI (14.9 vs. 25.4%). In addition, PSI is associated with a lower body mass index, plasma fasting blood glucose, postprandial blood glucose, glycated hemoglobin A1c, and homeostatic model assessment-insulin resistance score (15). In one study, compared to those in the control group (34.01%), the prevalence of metabolic syndrome (18.28%) and its components including central obesity, hypertriglyceridemia, and low high-density lipoprotein cholesterol were lower in the PSI group (16). Based on these findings, we aimed to provide a comprehensive review summarizing the detailed immune and metabolic regulatory mechanisms associated with *Schistosoma* infection and *Schistosoma*-derived products, as they relate to the treatment of T2D. We comprehensively describe the role of each type of immune cell in the modulation of host metabolism and immunity by *Schistosoma* infection and *Schistosoma*-derived molecules.

## Innate Immune System

Elements of the immune system such as acute phase reactants contribute to the development of T2D. AT inflammation mediates IR via dynamic immune cell regulation. AT contains most types of innate immune cells that exacerbate IR, including classically activated macrophages (CAMs), mast cells, and neutrophils. However, this tissue also contains eosinophils, alternatively activated macrophages (AAMs), dendritic cells (DCs), and innate lymphoid cells, which appear to have protective effects against T2D. In addition to *Schistosoma* infection *Schistosoma*-derived molecules might dampen inflammation and improve glucose tolerance by changing the immune cell composition in AT.

The first immune cell to be identified in AT was the AT macrophage (ATM). Macrophages are a very diverse group of cells: they are functionally and morphologically distinct in different tissues, and even in the same tissue, there are many different subpopulations of macrophages. Despite this diversity, macrophages are often classified as M1 (CAMs) and M2 (AAMs). M1 and M2 cells are distinct in terms of phenotype and function. M2 macrophages express CD11b, F4/80, CD301, and CD206, whereas M1 macrophages express CD11c, CD11b, and F4/80. M2 macrophages are recruited and retained in AT and participate in the regulation of immune cell populations in lean tissue. By contrast, the M1/M2 cell balance shifts toward the M1 phenotype during obesity, and these cells are primarily found in a ring-like configuration surrounding large adipocytes undergoing cell death. *In vivo*, macrophages comprise a heterogeneous population and can exhibit diverse phenotypes from anti- to pro-inflammatory. Furthermore, ATMs display plasticity and can alter or “switch” phenotypes in response to changes in the local microenvironment (17). A recent study demonstrated that *S. japonicum* infection promotes macrophage differentiation into the M2 phenotype and suppresses lipopolysaccharide (LPS)-induced activation of M1 cells. Furthermore, soluble egg antigen (SEA) from *S. japonicum* was shown to have the same effect as whole worm infection, except that it did not affect M1 macrophages (18). *Schistosoma*-derived lysophosphatidylcholine triggers the AAMs/M2 profile through increased expression of arginase-1, which is considerably inhibited by the peroxisome-proliferator-activated receptor γ (PPAR-γ) antagonist GW9662 (19). Sm 16, which is a major component of a *S. mansoni* cercarial product, prevents macrophage classical activation and delays antigen processing (20). Hussaarts et al. (21) reported that chronic *S. mansoni* infection in high-fat diet (HFD)-induced obese C57BL/6 male mice reduced adipocyte size and IR and improved peripheral glucose uptake and insulin sensitivity. Analysis of immune cell composition by flow cytometric (FCM) analysis and RT-PCR revealed that *S. mansoni* infection induces a considerable increase in AT M2 cells.

Dendritic cells are the primary professional antigen-presenting cells of the immune system (22). The primary cell-surface marker used to identify DCs, based on FCM analysis, is CD11c, although CD80, CD83, and CD86 are also commonly used in combination with other markers. Two studies sought to determine the role that DCs play in the development of obesity-induced IR, demonstrating that obesity results in increased DC numbers in AT (23, 24). Furthermore, DCs in obese subjects are functional and might be important regulators of AT inflammation in obesity-associated IR. Seifarth et al. (25) reported that a hyperglycemic environment affects the pool of peripheral DCs, leading to a reduction in the number of both myeloid DCs and plasmacytoid DCs. In the case of bone marrow-derived DCs, *S. mansoni* antigens polarize these cells, resulting in a more immature phenotype, and promoting Th2-type responses *in vivo*. Murine DCs are characterized by high-level expression of integrin CD11c. Diphtheria toxin depletion in CD11c^+^ cells results in dramatically impaired CD4^+^ T cell production of Th2 cytokines either after *S. mansoni* egg injection or during active *S. mansoni* infection, resulting in increased IFN-γ production and a shift toward a Th1 response (26). *Schistosoma*, and especially the eggs, comprises the strongest Th2-driving stimulus; furthermore, SEA-pulsed DCs can prompt Th2 differentiation in cocultured T cells *in vitro* (27). DCs also promote interleukin 10 (IL-10)-producing type 1 regulatory T cells (Tr1), through the action of *Schistosoma* lysophosphatidylserine, to induce regulatory responses (28). Two independent studies reported phenotypic and functional changes in DCs exposed to the *Schistosoma* antigen omega-1 (ω-1), which promotes the induction of a Th2 response in these cells (29, 30). ω-1 modulates the inflammatory effects induced by LPS in human monocyte-derived DCs and in murine bone marrow-derived cells. By contrast, depletion of ω-1 in SEA abrogates its Th2-inducing effects *in vitro*.

Eosinophils are multifunctional leukocytes that reside in certain organs and tissues, such as the intestine, blood, mammary glands, and AT. Eosinophils play an important role in metabolism, and they regulate macrophage activation state in mammalian AT. Previous experimental studies have shown a negative association between T2D and eosinophils, as IR was shown to play an important intermediate role in the relationship between eosinophils and impaired glucose metabolism. Studies on IL-4 in obesity suggest that eosinophils participate in the development of obesity-induced IR (31). Analyses of IL-4-green fluorescent protein mice (4get reporter mice) fed normal chow revealed that approximately 90% of IL-4-producing AT immune cells were eosinophils, although the number of AT eosinophils was very low (approximately 20,000/g of fat). Furthermore, obesity was shown to decrease AT eosinophil number. Zhu et al. (32) reported an association between the percentage of peripheral eosinophils and impaired glucose metabolism/IR in a large population, and demonstrated that a higher percentage of peripheral eosinophils are associated with a decreased risk of T2D. Furthermore, the authors demonstrated that eosinophil-knockout mice, generated from dblGATA mice, exhibited exacerbated obesity-induced IR, whereas a higher number of eosinophils, due to overexpression of IL-5 or helminth infections, improved obesity-induced IR (33). Increased eosinophil levels are a hallmark of chronic helminth infection, and there is substantial evidence that eosinophils play an immunoregulatory role in the protection against T2D. In mice infected with *S. mansoni*, eosinophils were shown to be an important cellular source of Th2-type cytokines, and in particular IL-4. In humans, a study of *S. mansoni* endemic populations in Uganda showed that eosinophils are associated with Th2 polarization, as indicated by IL-5 production, and can be a source of Th2-type cytokines. In fact, levels of Th2 cytokines (IL-4, IL-5, and IL-13) produced in response to *S. mansoni* adult worm antigen (SWA) *in vitro* were highest in cultures of human peripheral blood mononuclear cells (PBMCs) alone, and considerably lower than those in the supernatants of PBMC + eosinophil cocultures; moreover, they were lowest in cultures of eosinophils alone. This study showed that eosinophils down-modulate the *Schistosoma*-specific Th2 cytokine response in *S. mansoni-*infected individuals (34). Hams et al. (35) reported that recombinant *S. mansoni* egg-derived ω-1 improves the metabolic status of obese mice, and that this is mediated by the release of cytokine IL-33, which initiates the recruitment of eosinophils in AT. Chronic *S. mansoni* infection and SEA injections induce a strong increase in eosinophil infiltration into AT and protect against metabolic disorders in low-fat diet (LFD)- and HFD-fed mice.

**Figure 1:**
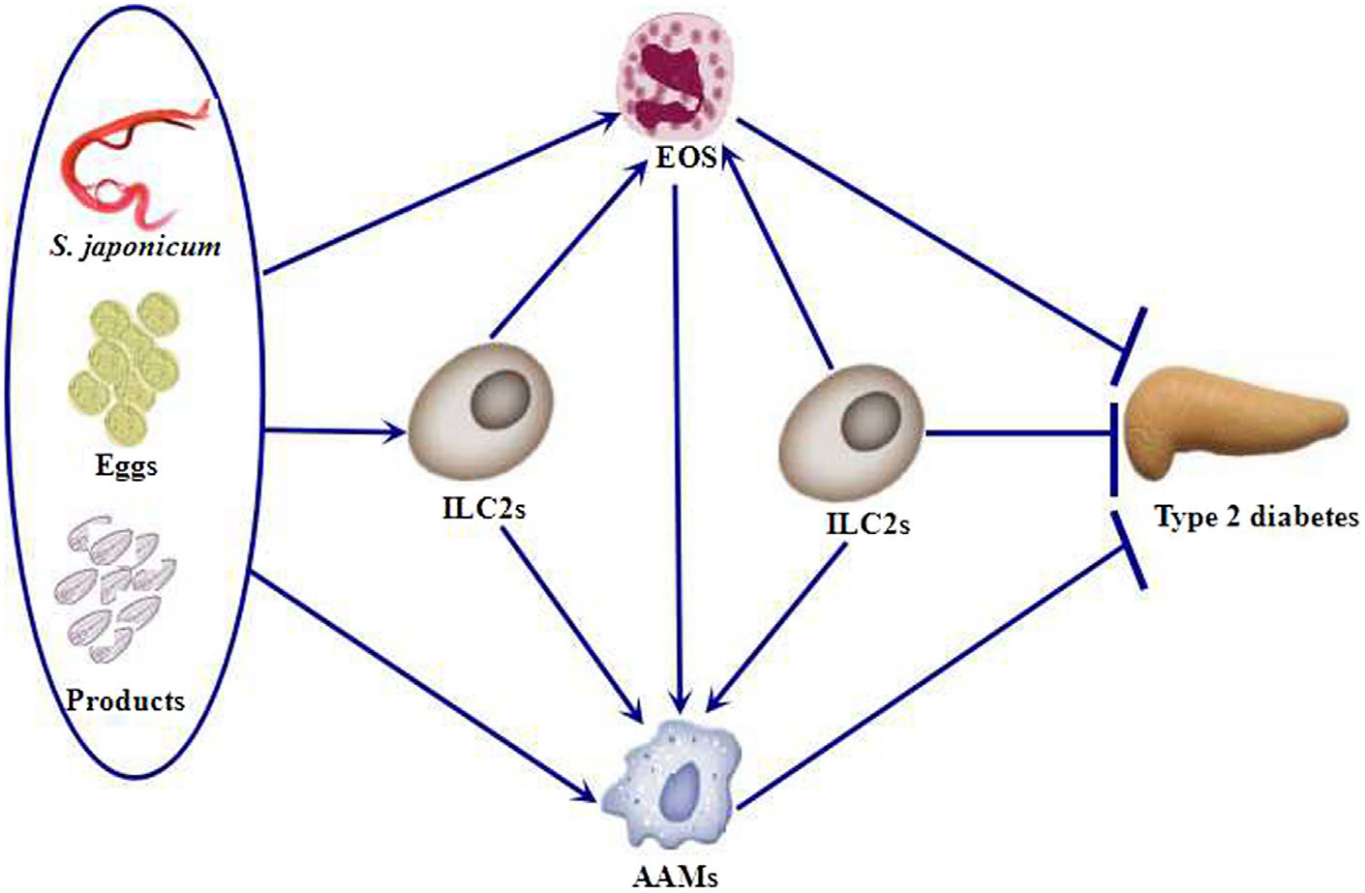
*Schistosoma* and *Schistosoma*-derived products induce the accumulation of ILC2s, eosinophils, and AAMs. ILC2s are also required to sustain the presence of eosinophils and AAMs. Eosinophils, AAMs, and ILC2s protect against type 2 diabetes (T2D). EOS, eosinophils; ILC2s, group 2 innate lymphoid cells; AAMs, alternatively activated macrophages.

Group 2 innate lymphoid cells (ILC2s) are innate cells that produce Th2 cytokines and are systemically distributed in many tissues including AT. During mouse and human development, ILC2s produce classical Th2 cytokines (IL-4, IL-5, and IL-13) in response to IL-25 and IL-33 (36). Secretion of Th2 cytokines in AT promotes tissue homeostasis and protects against obesity-induced metabolic dysfunction and T2D (37). Through the functional deletion of ILC2s, Molofsky et al. demonstrated that these cells are required to sustain the presence of eosinophils and M2 macrophages in AT, as they are the major source of Th2 cytokines (38). Nausch et al. (39) reported that the frequencies of ILC2s are diminished in young children (6–9 years old) infected with *S. haematobium* and are restored after removing the parasites. These data indicate that the association between a human parasitic infection and ILC2s has an important role before the establishment of protective acquired immunity during human schistosomiasis. This is due to a reduction in the generation and/or maintenance of cells after *S*. *haematobium* parasite infection. In fact, ILC2s might be recruited to the site of infection or to the tissues containing trapped eggs, initiating a localized immune response that leads to a reduction of cells in the peripheral blood. *S. mansoni*-SEA and *S. mansoni* egg-derived-ω-1 antigen administration enhances the number of ILC2s in AT and results in slightly increased IL-5 production. Recently, Hams et al. (35) demonstrated that ω-1 induces production of the cytokine IL-33, which induce ILC2s. They further showed that ω-1 fails to induce the infiltration of eosinophils and M2 macrophages into AT in the absence of ILC2s. Therefore, the ILC2–eosinophil–AAM axis is central to the maintenance of AT insulin sensitivity. We summarize the relationship among this axis, *Schistosoma*, and T2D (Figure 1).

## Acquired Immune System

**Figure 2:**
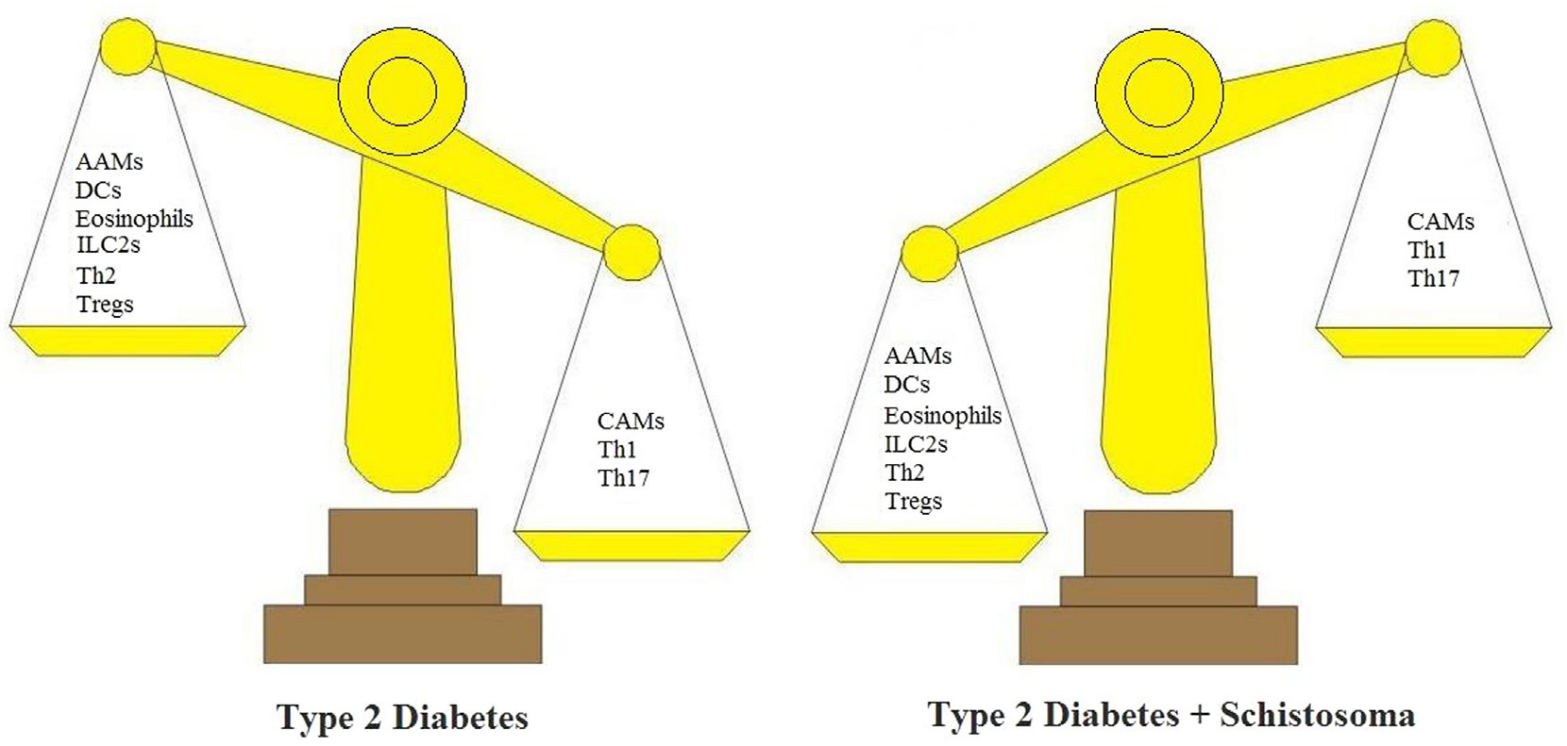
*Schistosoma* modulates the immunologic balance of type 2 diabetes (T2D). In T2D, immunologic elements are associated with inflammatory responses. These include CAMs, Th1 cells, and Th17 cells. In T2D + *Schistosoma*, immunologic elements include AAMs, DCs, ILC2s, Th2, and Tregs. AAMs, alternatively activated macrophages; CAMs, classically activated macrophages; DCs, dendritic cells; ILC2s, group 2 innate lymphoid cells; Th1, T helper 1 cells; Th2, T helper 2 cells; Tregs, regulatory T cells.

Helper T cells can be first divided into Th1 and Th2 cells according to the production of different cytokines. Th1-type cytokines include IL-12, IFN-γ, and TNF-α. However, Th1/Th2 is insufficient to explain this classification; many additional Th subsets including IL-17-producing Th17 cells, Tregs, and recently, IL-9-producing Th9 cells and IL-22-producing Th22 cells have been discovered. *Schistosoma* infection and *Schistosoma* products can effectively treat T2D by increasing Th2 T cell production and possibly Treg levels, while decreasing Th1 and Th17 T cell levels (Figure 2).

IFN-γ is a signature cytokine secreted from Th1 cells, which is pathogenic for the development of glucose intolerance and IR. IFN-γ mRNA expression is positively correlated with markers of obesity and glucose tolerance in T2D patients (40). Previous research demonstrated that IFN-γ-induced sustained loss of insulin-stimulated glucose uptake in human adipocytes, concurrent with reduced serine/threonine kinase phosphorylation and downregulation of the insulin receptor, was likely mediated by sustained JAK–STAT1 pathway activation (41). Thus, it is not surprising that IFN-γ deficiency protects obese mice from glucose intolerance and IR (42). T-bet, a major transcriptional regulator of IFN-γ, plays an important role in the development of IR in response to HFD; this provides further confirmation for the importance of this cytokine, as the absence of T-bet protects against the development of IR (43). IL-12, a cytokine that promotes Th1 differentiation and elevations in IFN-γ, has also been reported to be elevated in T2D patients (44). In *Schistosoma*-infected mice, the Th1-type immune response was predominant in the early stages. Subsequently, this switches to a Th2 immune response after the deposition of eggs. In fact, protection from infection was related to both Th1 and Th2 gene polymorphisms, indicating that Th1- and Th2-derived components contribute to immune responses to *Schistosoma* (45). It has been reported that lung-stage *Schistosoma* antigens are potent inducers of Th1 cellular immune responses and that vaccination with recombinant small heat-shock protein 70 from *S. japonicum* (rSjHSP70) induces a mixed Th1/Th2-type antibody response against SjHSP70 (46). A Th1-type response is pathogenic during T2D, and the enhanced Th2-type response resulting from *Schistosoma* infection changes the Th1/Th2 dichotomy during the development of T2D.

Type 2 helper T cells secrete the cytokines IL-4, IL-5, IL-13, IL-9, and IL-10. A study on overweight/obese human subjects showed that Th2 frequency in AT was inversely correlated with C-reactive protein levels, a marker of systemic inflammation, whereas Th1 frequency in these tissues was directly correlated with plasma concentrations of CRP. Th2 frequencies in both AT depots and peripheral blood were inversely associated with systemic IR (47). Administration of IL-4 to mice with diet-induced obesity (DIO) protects them from weight gain and glucose intolerance, and this involves activation of the STAT6 pathway (48). Adipocytes represent another important source of IL-4 and IL-13; these are also well known for their ability to induce M2 macrophages, which are protective against glucose intolerance. Analyses of the immune response induced by *Schistosoma* infection have revealed the development of a prominent Th2 response at the time of egg production during primary infection (approximately 6 weeks post-exposure). Furthermore, isolated *Schistosoma* eggs and SEA are able to directly induce Th2 responses when they are injected into mice (49). In addition, glycoproteins found in SEA, such as ω-1 (a T2 RNase), are capable of mediating immunomodulatory effects (50). Lacto-N-fucopentaose III, containing some Lewis^x^ motifs that are highly abundant in SEA, also induced a type 2 immune response (51). *Schistosoma* products are associated with several mechanisms that skew the Th2 response. One is signal dependent, whereas the other depends on its RNase activity. For example, ω-1 permits the cleavage of both ribosomal and messenger RNA to skew the immune response toward a Th2 profile. When previously unexposed travelers are infected with *Schistosoma*, they show an acute inflammatory immune response against the parasite, with large granulomatous reactions around the deposited eggs. However, during chronic infection, inflammatory cytokine responses to SEA are reduced, while Th2 cytokines are upregulated, and only a small proportion of patients progress to severe hepatosplenic disease (52). Hussaarts et al. investigated whether *S. mansoni*-derived molecules alleviate diet-induced metabolic disorders. With repetitive intraperitoneal injections of SEA, administration of a HFD to mice promotes the development of Th2 cells through enhanced expression of OX40 ligand; this was shown *in vitro* (53) and *in vivo* (54). Importantly, SEA treatment for 4 weeks improved fasting plasma glucose and insulin levels, whole-body glucose tolerance, HOMA-IR, and insulin sensitivity. In obese animals, induction of a type 2 cellular response in AT leads to weight loss and improved glucose homeostasis. Injecting obese mice with recombinant *S. mansoni* egg-derived ω-1 improves metabolic status, which involves a mechanism reliant upon the release of the type 2 initiator cytokine, IL-33.

Regulatory T cells play an important role in the suppression of inflammation. These cells are abundant in the AT of lean mice, but their number is greatly reduced in IR animal models of obesity (55). Loss-of-function and gain-of-function experiments have revealed that Tregs affect inflammation in AT and, thus, IR. *In vivo*, inducing the general development of Tregs with IL-2/anti-IL-2 complexes significantly improves insulin sensitivity in obese mice. Similarly, adoptive transfer of CD4^+^ T cells expressing GATA binding protein 3 normalizes IR (56). Conversely, after Treg depletion in Foxp3–diphtheria toxic receptor transgenic mice, IR develops spontaneously, and insulin signaling in insulin-responsive tissues including AT is impaired. Tregs exert their regulatory effects in many ways, often through the secretion of IL-10 or transforming growth factor β (TGF-β), which block the production of inflammatory cytokines or counteract the TNF-α-mediated inhibition of insulin signaling in adipocytes. There is evidence suggesting that IL-10 is involved in T2D-related inflammation as mice engineered to ectopically express IL-10 (*via* gene transfer) are partially protected from HFD-induced obesity and glucose intolerance (57). In addition, overexpression of IL-10 improves insulin sensitivity. Tregs expressing TGF-β-dependent latency-associated peptide were found to be induced by orally administered anti-CD3 antibody and β-glucosylceramide combination therapy, which helped to reduce IR, fat accumulation in the liver, and inflammation in AT, and this was accompanied by lower blood glucose and liver enzymes in leptin-deficient ob/ob mice (58). In the 1970s, Ottesen et al. (59) first reported cellular immune hyporesponsiveness in individuals infected with helminths. Results indicated that lymphocytes isolated from subjects chronically infected with *S. mansoni* showed a diminished proliferative response upon stimulation with *Schistosoma* antigens. Patients presented with high percentages of Tregs, which declined after effective treatment with praziquantel. Furthermore, Treg phenotypes changed upon treatment, with fewer Tregs expressing CD45RO, a marker associated with T-cell memory and suppressive activity (60). My own work has further confirmed the positive association between Tregs and cellular immune hyporesponsiveness. Tregs were shown to contribute to evasion of the host immune response by *S. japonicum*, whereas an anti-CD25 monoclonal antibody (mAb) partially blocked Tregs and enhanced protective immunity to the parasite *via* a Th1-type immune response (61). Although Tregs have been extensively investigated in animal models and human subjects with autoimmunity and type 1 diabetes (T1D), the induction of Tregs to treat T2D has not been performed, and the exact role of Tregs (subsets) in AT insulin sensitivity is still a matter of debate (62). Therapies that specifically increase Tregs might be useful for treating IR (63). One of the current drugs for T2D, pioglitazone, can improve insulin sensitivity by stimulating PPARγ signaling in Tregs, leading to increased AT Treg cell frequency (64). Recently, Surendar et al. hypothesized that helminths prevent T1D onset by mitigating pancreatic inflammation, conferring protection against T2D by improving insulin sensitivity, alleviating inflammation, augmenting AT, and improving lipid metabolism and insulin signaling (65).

Although, more recently, other cells were also shown to express IL-17, including γδT cells, natural killer T, and macrophages, Th17 (type 17 helper T cells) are the major source of IL-17 (66). It is well established that IL-17-producing Th17 cells exacerbate autoimmune and inflammatory diseases. In line with the hypothesis that Th17 cells are pathogenic during T2D, diabetic patients display an increased frequency of peripheral blood Th17 cells compared to that in non-diabetic controls. In addition, levels of IL-17 and IL-6, which are known to induce Th17 differentiation, are positively correlated with the severity of diabetes. AT from metabolically abnormal insulin-resistant obese (MAO) subjects was shown to exhibit 3- to 10-fold increases in the number of CD4^+^ T cells that produce IL-22 and IL-17, as compared to levels in metabolically normal insulin-sensitive obese subjects and lean subjects. MAO subjects produce cytokines that cause metabolic dysfunction in the liver and muscle *in vitro* (67). IL-17 is a pro-inflammatory cytokine involved in the pathogenesis of many inflammatory and infectious conditions including schistosomiasis. In addition, elevated Th17 levels were observed in response to vaccination against *S. mansoni* infection in C57BL/6 mice (68). Furthermore, *Schistosoma*-induced downregulation of Th1 and Th17 cells occurs after infection, at the stage comprising the beginning of egg-laying. This result suggests that egg deposition is the major stimulus for the dampening of Th17 responses, indicating that *S. japonicum* egg antigens, but not adult worm antigens, preferentially inhibit Th17 cell generation. Furthermore, blocking IL-17 with a neutralizing mAb increases *Schistosoma*-specific antibody levels and confers partial protection against *S. japonicum* infection in mice (69).

A recent study showed that obesity increases the B cell population in AT, and that B lymphocytes are involved in inflammation and are related to IR during obesity and T2D (70). Conversely, the depletion of B cells by a neutralizing antibody improves obesity-induced IR. To investigate the effect of B cells on IR, DIO B cell knockout mice (μMT mice) were studied, and improved glucose tolerance and insulin sensitivity were detected. B cell deficiency was associated with a reduction in TNF-α-producing M1 macrophages and activated CD8^+^ T cells in AT. Similarly, mice treated with a CD20-specific B cell depleting antibody had fewer TNF-α^+^ macrophages in their AT (71). DeFuria et al. (72) confirmed that μMT mice have lower serum pro-inflammatory cytokine levels and increased Tregs in the spleen and AT. B cells promote a specific pro-inflammatory T-cell ratio during obesity, which is characterized by the same increase in Th17 and Th1 functions and decrease in Treg numbers that are identified in T2D patients (73). The presence of suppressive B cells, similar to Tregs in terms of their ability to suppress inflammation and the secretion of the anti-inflammatory cytokine, IL-10, has also been demonstrated. These are known as regulatory B cells (Bregs). Suppressive B cells that secrete IL-10 are essential for suppression during murine infections by *S. mansoni*. Furthermore, the transfer of *S. mansoni*-induced Bregs induces the recruitment of Tregs to inflammatory airways in an IL-10-dependent manner. These results were replicated in mice lacking the ability to undergo antibody class switch. A role for antibodies in the prevention of severe diseases has also been suggested based on the finding that pulmonary involvement is also apparent in mice unable to secrete class-switched antibodies. One major effect of anti-IL-10R treatment is the loss of an IgG1^+^ myeloid population that exhibits characteristics of regulatory/anti-inflammatory macrophages. The data indicate a role for IL-10-dependent B cell responses in the regulation of tissue damage during chronic helminth infection (74). In summary, both antibody production and immunoregulatory cytokine production by B cells are important for immunomodulation during chronic *Schistosoma* infections.

## Conclusion

The application of helminth parasites or their subunits for disease treatment has been suggested by the hygiene hypothesis (75), in which purified or synthetic immunomodulatory products from worms have been considered for clinical purposes. Parasite infection and products from parasites have major *in vivo* effects in ameliorating T2D and inflammatory disorders; however, their medical application might be limited by the adverse effects of *Schistosoma* infection and the potential immunogenicity of their products. Various immunomodulatory molecules (carbohydrates, proteins, and lipids) have been identified, such as LNFP III, which are capable of inducing B cells (especially B-1 cells) to secrete IL-10. LNFP III was also reported to alternatively activate macrophages. The identification of single active molecules and the study of the mechanisms through which they improve whole-body metabolic homeostasis might offer new insights into the development of novel therapeutics for the treatment of T2D.

## Author Contributions

C-lT is mainly responsible for literature collection and paper writing. Z-ML is responsible for collecting the references. YG is mainly responsible for literature collection and paper writing. FX is corresponding author who is in charge of directing the paper.

## Conflict of Interest Statement

The authors declare that the research was conducted in the absence of any commercial or financial relationships that could be construed as a potential conflict of interest.
